# Extracellular Vesicle-Mediated Communication in Sellar Tumors: A Conceptual and Translational Framework

**DOI:** 10.3390/ijms27114947

**Published:** 2026-05-29

**Authors:** Pierlorenzo Veiceschi, Giuseppa D’Amico, Giovanni Tringali, Francesco Cappello, Celeste Caruso Bavisotto, Alessandra Maria Vitale

**Affiliations:** 1 Neurosurgery Department, ARNAS Civico Di Cristina Benfratelli Hospital, Piazza Leotta 4, 90127 Palermo, Italy; giovanni.tringali@arnascivico.it; 2 Department of Biomedicine, Neuroscience and Advanced Diagnostics (BIND), University of Palermo, Via Del Vespro 129, 90127 Palermo, Italy; giuseppa.damico01@unipa.it (G.D.); francesco.cappello@unipa.it (F.C.); celeste.carusobavisotto@unipa.it (C.C.B.); 3 Euro-Mediterranean Institute of Science and Technology (IEMEST), Via Michele Miraglia, 90139 Palermo, Italy

**Keywords:** extracellular vesicles, exosomes, sellar tumors, pituitary neuroendocrine tumors, craniopharyngioma, tumor cells-derived EVs, MicroRNAs, non-coding RNAs, liquid biopsy, biomarkers

## Abstract

Sellar tumors, including pituitary neuroendocrine tumors (PitNETs), craniopharyngiomas, and rare malignant entities, represent a heterogeneous group of lesions characterized by complex interactions within the tumor microenvironment (TME). In recent years, extracellular vesicles (EVs) have emerged as key mediators of intercellular communication, playing a crucial role in tumor progression, invasion, and therapeutic resistance across multiple cancer types. Despite growing interest in EV biology, their role in sellar tumors remains poorly defined due to limited direct experimental evidence. This review provides a conceptual and translational framework for understanding EV-mediated communication in the context of sellar region tumors. We summarize current knowledge on EVs biogenesis, molecular cargo, and mechanisms of uptake, and discuss how these vesicles may influence tumor behavior through modulation of proliferation, angiogenesis, extracellular matrix (ECM) remodeling, and immune responses. Particular attention is given to PitNETs and craniopharyngiomas, integrating available evidence with insights derived from related tumor models. We further explore the potential of EVs as minimally invasive biomarkers and therapeutic tools and highlight the major technical and biological challenges that currently limit clinical translation. By identifying key knowledge gaps and future research directions, this review aims to guide further investigation into the role of EVs in sellar tumor biology.

## 1. Introduction

Sellar tumors represent a heterogeneous group of lesions arising in the pituitary region, including pituitary neuroendocrine tumors (PitNETs), craniopharyngiomas, and rare malignant entities [1,2,3]. Although most of these tumors are histologically benign, their clinical behavior can be highly variable, ranging from indolent growth to aggressive and invasive forms associated with significant morbidity [4,5]. In particular, tumor invasion into surrounding structures such as the cavernous sinus, as well as recurrence after surgical or medical treatment, remain major clinical challenges.

Current diagnostic work-up for sellar tumors relies on the integration of high-resolution magnetic resonance imaging (MRI), endocrine biochemical assessment, ophthalmological evaluation, and, when available, histopathological and molecular characterization. MRI remains essential for defining tumor size, extension, cavernous sinus invasion, optic pathway compression, and surgical planning, whereas hormonal profiling is critical for classifying functioning lesions and monitoring treatment response. However, despite advances in neuroimaging, surgical techniques, and pharmacological therapies, reliable biomarkers for predicting tumor behavior and guiding personalized management are still lacking, as current approaches remain limited: imaging cannot always predict biological aggressiveness or recurrence risk, endocrine markers are unavailable for non-functioning lesions, ophthalmological assessment reflects functional impairment rather than tumor biology, and histopathology is usually obtained only after surgery [6].

In recent years, increasing attention has been directed toward the role of the tumor microenvironment (TME) in shaping the biological behavior of sellar tumors. Beyond intrinsic genetic and epigenetic alterations, tumor progression is now recognized as a dynamic process driven by complex interactions between neoplastic cells and surrounding stromal, vascular, and immune components [7]. These interactions are mediated by a variety of signaling mechanisms, including soluble factors, direct cell–cell contact, and, more recently, extracellular vesicles (EVs) [8].

EVs are membrane-bound particles released by virtually all cell types and are now considered key mediators of intercellular communication [9,10]. By transferring a diverse molecular cargo—including microRNAs (miRNAs), proteins, lipids, and nucleic acids—EVs are able to modulate the phenotype and function of recipient cells, thereby influencing multiple aspects of tumor biology [11]. In several cancer types, EVs have been implicated in promoting tumor growth, angiogenesis, immune evasion, extracellular matrix (ECM) remodeling, and metastatic dissemination [12]. Furthermore, their presence in biological fluids has raised considerable interest in their potential use as minimally invasive biomarkers and therapeutic tools [13].

According to the most recent Minimal Information for Studies of Extracellular Vesicles (MISEV2023) recommendations, the term “extracellular vesicles” is preferentially used throughout this review as an operational umbrella term, unless the original studies specifically characterized exosomes or other EV subtypes using appropriate criteria. Accordingly, terms such as “exosomes”, “small EVs”, “serum EVs”, “tumor-derived EVs”, or “cyst-fluid EVs” are used only when they reflect the nomenclature, biological source, or methodological characterization reported in the cited studies [10].

Despite the growing body of evidence supporting a central role of EVs in cancer biology, their contribution to the pathophysiology of sellar tumors remains poorly defined [14]. The relative rarity of these tumors, combined with their biological heterogeneity, has limited the availability of direct experimental and clinical data. As a result, current knowledge is largely fragmented, and the potential implications of EV-mediated communication in this context are likely underappreciated [15]. EV-mediated communication represents an emerging layer of regulation that may partially explain the discrepancy between histological benignity and clinical aggressiveness of sellar tumors.

In this review, we aim to provide a comprehensive and integrative overview of extracellular vesicle-mediated communication in sellar tumors, with a particular focus on microenvironment interactions.

By synthesizing available evidence from pituitary and non-pituitary models, this review proposes a conceptual and translational framework for understanding EV-mediated communication in sellar tumor growth, invasiveness, microenvironmental remodeling, and therapeutic response. We further discuss the emerging role of EVs as diagnostic, prognostic biomarkers and as potential tools for targeted therapies, while outlining current limitations and future research directions. Importantly, experimentally validated findings in sellar tumors are distinguished from hypothesis-generating interpretations extrapolated from neuro-oncological, endocrine, or other solid tumor models, acknowledging that mechanisms established in other cancers may not fully reflect the anatomical, endocrine, and microenvironmental specificity of sellar lesions.

**Figure 1:**
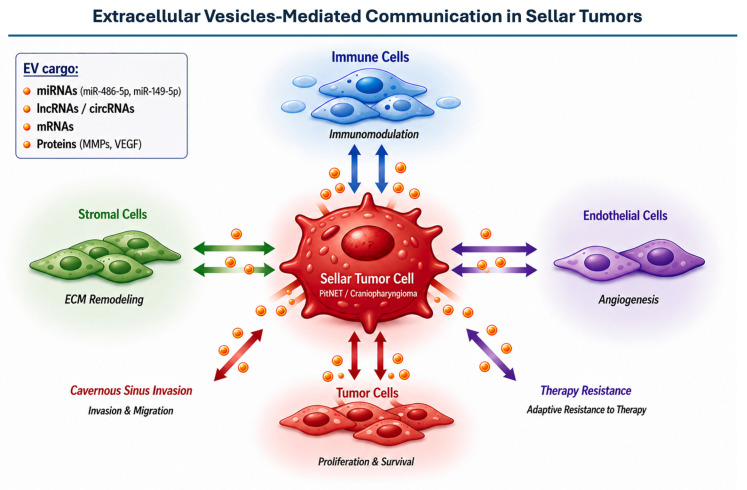
Conceptual framework of EVs-mediated communication in sellar tumors. These vesicles mediate intercellular communication with tumor cells (autocrine/paracrine signaling), stromal cells, endothelial cells, and immune cells, thereby influencing key biological processes including proliferation, ECM remodeling, angiogenesis, immune modulation, invasiveness, and therapy resistance.

A conceptual overview of extracellular vesicle-mediated communication in sellar tumors and its potential biological and clinical implications is illustrated in Figure 1.

## 2. Extracellular Vesicles in Tumor Biology

EVs are a heterogeneous population of membrane-bound particles released by virtually all cell types under both physiological and pathological conditions [9]. Historically, EVs have been classified into exosomes, microvesicles, and apoptotic bodies according to their proposed biogenesis and size range. However, because size, density, surface markers, and biogenetic pathways overlap substantially, current MISEV recommendations encourage the use of operational terminology based on measurable physical characteristics, biochemical composition, cellular source, or isolation method rather than assuming a specific biogenesis when this has not been directly demonstrated. This distinction is particularly relevant when interpreting studies on circulating or cyst-fluid EVs, in which mixed vesicle populations are commonly analyzed [10].

Exosomes originate from the endosomal pathway through the inward budding of the limiting membrane of multivesicular bodies, which subsequently fuse with the plasma membrane to release their intraluminal vesicles into the extracellular space [16]. This process is regulated by both endosomal sorting complex required for transport (ESCRT)-dependent and ESCRT-independent mechanisms, involving proteins such as ALIX, TSG101, and tetraspanins [17]. In contrast, microvesicles are generated by the outward budding of the plasma membrane, a process driven by cytoskeletal rearrangements and changes in membrane lipid composition. These distinct biogenetic pathways contribute to differences in cargo composition and functional properties.

The molecular cargo of EVs reflects, at least in part, the cellular origin and physiological state of the releasing cells. EVs carry a wide range of bioactive molecules, including proteins, lipids, messenger RNAs (mRNAs), miRNAs, long non-coding RNAs (lncRNAs), and, in some cases, fragments of genomic and mitochondrial DNA [11]. Importantly, cargo loading is not a passive process but is selectively regulated, enabling EVs to function as targeted delivery systems for specific signaling molecules. In the context of cancer, tumor cells-derived EVs (TCdEVs) are often enriched in oncogenic proteins, pro-angiogenic factors, and regulatory RNAs that can reprogram recipient cells [18].

Once released into the extracellular space, EVs can interact with target cells through multiple mechanisms [19]. These include direct membrane fusion, receptor–ligand interactions, and various forms of endocytosis, such as clathrin-mediated uptake, macropinocytosis, and phagocytosis [20]. Through these processes, EVs deliver their molecular cargo to recipient cells, thereby modulating gene expression, signaling pathways, and cellular behavior [21]. This capacity to transfer functional biomolecules underlies their role as key mediators of intercellular communication.

In cancer biology, EVs have emerged as critical regulators of tumor progression and microenvironmental remodeling [8]. TCdEVs can promote proliferation by transferring oncogenic signals, enhance angiogenesis through the delivery of pro-angiogenic factors, such as the vascular endothelial growth factor (VEGF), and facilitate ECM degradation via matrix metalloproteinases (MMPs) [22]. Moreover, EVs play a pivotal role in immune modulation, contributing to immune evasion by suppressing anti-tumor immune responses or by inducing tolerogenic phenotypes in immune cells [23]. Another important function of EVs is their involvement in the establishment of pre-metastatic niches. By conditioning distant tissues through the transfer of specific molecular cargo, EVs can create a permissive environment for tumor cell colonization [24,25]. Although metastatic spread is rare in most sellar tumors, similar mechanisms may contribute to local invasiveness, particularly in anatomically complex regions such as the cavernous sinus.

In addition to their functional roles, EVs have attracted considerable interest as potential biomarkers due to their stability in biological fluids, including blood and cerebrospinal fluid (CSF) [26]. Their molecular content can provide insights into tumor presence, subtype, and biological behavior, making them promising candidates for liquid biopsy approaches. Furthermore, their intrinsic ability to deliver cargo has positioned EVs as potential therapeutic vehicles, either by exploiting native vesicles or by engineering them for targeted drug delivery [27]. Notably, EVs can cross the blood–brain barrier (BBB), highlighting their potential as drug delivery systems in the context of brain tumors [28]. These mechanisms are likely conserved in sellar tumors, although evidence remains limited.

Despite these advances, several challenges remain in EV research. The heterogeneity of EV populations, the lack of standardized isolation and characterization protocols, and the difficulty in distinguishing between vesicle subtypes continue to limit reproducibility and clinical translation [29]. These issues are particularly relevant in the study of less common tumor types, such as those arising in the sellar region, where available data are still scarce. Overall, EVs represent a complex and highly dynamic system of intercellular communication with profound implications for tumor biology. Understanding their biogenesis, cargo selection, and mechanisms of action is essential to elucidate their role in disease processes and to harness their potential for theragnostic.

## 3. Tumor Microenvironment in Sellar Tumors

The TME has emerged as a critical determinant of tumor behavior, influencing growth dynamics, invasiveness, and response to therapy [7,30]. In sellar tumors, including PitNETs and craniopharyngiomas, the interaction between neoplastic cells and their surrounding microenvironment plays a particularly relevant role due to the anatomical and functional complexity of the sellar region [31]. The proximity to critical structures, combined with the absence of a true anatomical barrier, facilitates tumor cells’ invasion towards adjacent intracranial tissues.

PitNETs are traditionally considered benign lesions; however, a subset of these tumors exhibits locally aggressive behavior, characterized by invasion of the cavernous sinus, sphenoid sinus, and surrounding dura [32,33]. Increasing evidence suggests that this invasive phenotype cannot be explained solely by intrinsic tumor cell properties, but rather reflects dynamic interactions with the ECM and stromal components [4]. Alterations in ECM composition, including increased expression of MMPs and changes in adhesion molecules, contribute to tissue remodeling and facilitate tumor infiltration [34]. In parallel, angiogenic processes, mediated by factors such as VEGF, support tumor growth and may further influence the local microenvironment [35].

Craniopharyngiomas represent a distinct model of TME interaction [36,37]. Adamantinomatous craniopharyngiomas are characterized by a highly inflammatory milieu, with abundant infiltration of immune cells and production of cytokines and chemokines. The cystic component of these tumors contains a complex mixture of proteins, lipids, and inflammatory mediators, suggesting an active exchange of signaling molecules within the TME [38]. These features make craniopharyngiomas especially suitable for studying paracrine communication mechanisms, including those mediated by EVs.

In addition to tumor cells and the ECM, multiple non-neoplastic cell populations contribute to the sellar TME. These include fibroblasts, endothelial cells, pericytes, and immune cells such as macrophages and lymphocytes. Tumor-associated macrophages, in particular, have been implicated in promoting tumor progression through the secretion of growth factors, proteases, and immunomodulatory molecules [39]. Similarly, endothelial cells play a key role in angiogenesis and vascular remodeling, processes that are essential for tumor expansion within the confined sellar space [40].

Although pituitary carcinoma is a rare entity, it provides insight into the extreme end of the biological spectrum of sellar tumors. The ability of these tumors to metastasize suggests the presence of molecular and microenvironmental mechanisms that go beyond local invasion, potentially involving systemic communication pathways. While direct evidence remains limited, these observations support the hypothesis that EV-mediated signaling may contribute to disease progression in more aggressive forms [41,42].

Overall, the sellar TME is a dynamic and multifaceted system in which cellular, molecular, and structural components interact to shape tumor behavior. Understanding these interactions is essential to identifying the mechanisms underlying tumor growth and invasiveness, and provides a conceptual basis for exploring the role of EVs as key mediators of communication within this complex ecosystem.

## 4. EVs in Sellar Tumors

EVs exert their biological effects primarily through the transfer of a diverse and functionally active molecular cargo [8,25]. In sellar tumors, although direct evidence remains limited, emerging data suggest that EV-associated cargo may play a relevant role in shaping tumor behavior and microenvironmental interactions [15]. The molecular components transported by EVs have the potential to influence multiple aspects of tumor biology, such as proliferation, invasiveness, angiogenesis, and immune modulation [43].

Importantly, the functional significance of EV cargo extends beyond tumor cells themselves, involving a dynamic exchange of signals between tumor and stromal compartments [25]. This bidirectional communication contributes to the establishment of a permissive microenvironment that supports tumor progression and adaptation to external stimuli [44]. In intracranial regions such as the sellar area, where tumor growth occurs in close proximity to critical neurovascular structures, these interactions may be particularly relevant in determining patterns of local invasion and clinical behavior [45,46].

The following sections will explore in detail the different categories of EV-associated cargo identified in sellar tumors. Particular attention will be given to their potential biological functions and clinical implications, integrating available evidence from sellar and related tumor models to provide a comprehensive and translational perspective. Where direct sellar tumor evidence is lacking, the proposed biological effects of EV cargo should be interpreted as mechanistic hypotheses requiring dedicated validation in PitNETs, craniopharyngiomas, or other sellar lesions. This is particularly important for processes such as immune modulation, angiogenesis, extracellular matrix remodeling, and therapy resistance, which are well described in other tumor models.

A summary of EV-associated molecular cargo and their proposed functional and clinical implications across sellar tumors is provided in Table 1.

### 4.1. EV-Associated miRNAs in Sellar Tumors

miRNAs are among the most extensively studied components of EV cargo and are considered key regulators of intercellular communication in cancer [8,11]. By modulating gene expression at the post-transcriptional level, EV-associated miRNAs can influence a wide range of biological processes, including cell proliferation, apoptosis, angiogenesis, and ECM remodeling [8,11,20,58]. In the context of sellar tumors, emerging evidence suggests that EV-derived miRNAs may contribute to tumor behavior and hold potential as diagnostic and prognostic biomarkers [58].

In PitNETs, several studies have identified distinct miRNA profiles within circulating EVs. In particular, differential expression of multiple exosomal miRNAs has been reported in patients with non-functioning pituitary adenomas compared to healthy controls. Among these, miR-486-5p has been proposed as a potential biomarker for tumor detection and disease monitoring, given its consistent upregulation in patient-derived EVs [47]. However, its association with tumor invasiveness remains unclear, as some studies have not demonstrated significant differences between invasive and non-invasive lesions. These findings highlight both the promise and current limitations of EV-associated miRNAs as clinical biomarkers [10].

Beyond diagnostic applications, EV-associated miRNAs may play a functional role in modulating tumor behavior. Experimental studies in pituitary tumor cell lines have shown that specific miRNAs, such as miR-149-5p and miR-99a-3p, are downregulated in TCdEVs. In particular, miR-99a-3p has been implicated in suppressing tumor progression by targeting components of the NOVA1-related regulatory axis, thereby interfering with downstream pathways involved in cell proliferation and vesicle trafficking [48]. Restoration of these miRNAs has been associated with reduced cellular proliferation, migration, and invasiveness, suggesting a tumor-suppressive role.

Additional layers of complexity arise from the heterogeneity of miRNA expression across different tumor subtypes and biological contexts. For instance, variations in circulating miRNA levels have been reported between invasive and non-invasive PitNETs, as well as among different hormonal subtypes [49]. However, the reproducibility of these findings remains limited, likely due to differences in study design, sample size, and EV isolation methodologies. This variability underscores the need for standardized approaches in EV research and highlights the challenges associated with translating miRNA-based biomarkers into clinical practice.

Although evidence remains limited, recent studies have demonstrated the presence of EVs within the cyst fluid of craniopharyngiomas, supporting their potential role in TME signaling [57]. In particular, miRNAs involved in inflammatory signaling, angiogenesis, and epithelial–mesenchymal transition (EMT) have been identified in EVs derived from various central nervous system and neuroendocrine tumors. By analogy, similar mechanisms may contribute to the inflammatory and cystic microenvironment observed in craniopharyngiomas, although this remains to be demonstrated experimentally.

### 4.2. EV-Associated lncRNAs and circRNAs in Sellar Tumors

In addition to miRNAs, EVs carry a diverse repertoire of non-coding RNAs, including lncRNAs and circular RNAs (circRNAs), which have emerged as important regulators of gene expression and tumor biology [8,20,59]. These RNA species are increasingly recognized for their ability to modulate transcriptional and post-transcriptional processes, often acting as competing endogenous RNAs that regulate miRNA availability and downstream signaling pathways [59].

In PitNETs, the study of EV-associated lncRNAs and circRNAs is still in its early stages, but initial evidence suggests their potential involvement in tumor progression and invasiveness [60]. Altered expression of lncRNAs has been reported in invasive pituitary adenomas, particularly in relation to bone invasion and aggressive growth patterns [48].

CircRNAs, due to their high stability and resistance to exonuclease degradation, are particularly well-suited for packaging into EVs and for detection in biological fluids. Emerging data indicate that specific circRNAs may be enriched in TCdEVs and contribute to tumor progression through miRNA sponging mechanisms [61]. For example, circDennd1b has been implicated in aggressive pituitary tumor behavior through modulation of the miR-145-5p/ONECUT2 axis and activation of downstream signaling pathways such as MAPK [53]. This circRNA has been described in EVs derived from tumor-associated fibroblasts [62]. In addition, EV-associated circCCDC66 has been proposed as a circulating exosomal biomarker with potential diagnostic value in PitNET [52].

The involvement of stromal cells in EVs production adds an additional layer of complexity to the regulatory network within sellar tumors. Cancer-associated fibroblasts, endothelial cells, and immune cells can release EVs enriched in lncRNAs and circRNAs that influence tumor cell phenotype, promoting proliferation, invasion, and resistance to therapy. This bidirectional exchange of RNA cargo between tumor and stromal compartments may play a critical role in shaping the TME and sustaining tumor progression [63].

Although direct evidence in craniopharyngiomas and other sellar lesions is limited, the known inflammatory and cystic characteristics of these tumors suggest that EV-mediated RNA transfer could be particularly relevant. The presence of protein- and lipid-rich cystic fluid raises the possibility that EVs carrying regulatory non-coding RNAs may contribute to local signaling networks, although this requires further investigation [57].

### 4.3. EV-Associated mRNAs and Proteins in Sellar Tumors

Beyond non-coding RNAs, EVs carry a variety of mRNAs and proteins that can directly influence the phenotype and functional state of recipient cells [8,20]. These components represent an additional layer of intercellular communication, enabling not only the modulation of gene expression through regulatory RNAs but also the transfer of bioactive proteins capable of altering signaling pathways in target cells.

In the context of sellar tumors, data on EV-associated mRNAs remain limited but suggest a potential role in tumor characterization and progression [15]. Circulating mRNA molecules encapsulated within EVs have been proposed as biomarkers reflecting tumor-specific transcriptional programs. For instance, Increased levels of neuroendocrine-related transcripts, such as Insulinoma-associated 1 mRNA (INSM1), have been suggested in translational analyses and summarized in recent studies, although direct validation in large clinical cohorts is still limited [64]. Although the functional impact of EV-mediated mRNA transfer in pituitary tumors has not been fully elucidated, evidence from other cancer models indicates that transferred mRNAs can be translated in recipient cells, thereby contributing to phenotypic reprogramming [50].

The protein cargo of EVs is even more linked to functional outcomes, as it includes enzymes, receptors, adhesion molecules, and signaling mediators that can rapidly influence cellular behavior. In PitNET, differential expression of specific proteins within circulating EVs has been observed between invasive and non-invasive lesions. Among these, epithelial and folate-related markers such as Epithelial cell adhesion molecule (EPCAM) and Folate receptor 1 (FOLR1) have been reported to be altered in EVs derived from patients with invasive tumors, suggesting a potential association with EMT-like processes and tumor invasiveness [43]. Recent proteomic analyses of exosomes isolated from craniopharyngioma cyst fluid have identified differential protein enrichment, particularly in lipid metabolism pathways, suggesting a role for EVs in shaping the TME [57].

EV-associated proteins may also contribute to ECM remodeling, a key step in tumor invasion. The presence of MMPs and other proteolytic enzymes within EVs has been widely documented in multiple tumor types and may facilitate local tissue degradation and tumor spread [65]. In the sellar region, where tumors must navigate complex anatomical boundaries such as the cavernous sinus and meningeal layers, EV-mediated delivery of proteases could represent a key mechanism for invasive growth [66].

In addition to proteases, EVs can carry pro-angiogenic factors, including VEGF and other signaling molecules that promote endothelial cell activation and neovascularization [67]. Angiogenesis is a relevant feature in pituitary tumors and may be partly driven by EV-mediated communication between tumor cells and endothelial cells. This interaction could enhance vascular permeability, support tumor expansion, and contribute to the establishment of a permissive microenvironment [68].

Another important aspect of EV protein cargo is its role in immune modulation. TCdEVs can carry immunosuppressive molecules capable of altering immune cell function, promoting tolerance, and inhibiting anti-tumor responses [69]. Although specific data on sellar tumors are scarce, the presence of immune cell infiltration in craniopharyngiomas and the emerging role of immune components in PitNETs suggest that EV-mediated immune regulation may be an underexplored but relevant mechanism [70].

Overall, EV-associated mRNAs and proteins provide functional insights into tumor biology that complement the regulatory roles of non-coding RNAs. Further studies integrating transcriptomic and proteomic analyses of EVs will be essential to clarify these mechanisms and to identify clinically relevant biomarkers [71].

### 4.4. Functional Implications of EV Cargo in Sellar Tumors

The biological relevance of EVs in sellar tumors is ultimately determined by the functional impact of their molecular cargo on recipient cells [8,11,25]. While individual components such as miRNAs, lncRNAs, mRNAs, and proteins provide mechanistic insights, their coordinated action drives key phenotypic changes, including tumor growth, invasiveness, microenvironmental remodeling, and therapeutic resistance [25,72,73].

EV-mediated signaling contributes to tumor cell proliferation and survival through the transfer of oncogenic miRNAs and activation of pathways such as PI3K/AKT and MAPK [73,74,75]. Conversely, the loss of tumor-suppressive miRNAs may promote uncontrolled growth, while their restoration in PitNET models has been associated with reduced proliferation and tumor cell viability [48].

EVs also play a relevant role in tumor invasiveness, particularly in PitNETs exhibiting cavernous sinus invasion, closely linked to ECM remodeling and alterations in cell adhesion [4,35]. EV-mediated delivery of MMPs and regulatory RNAs may modulate cytoskeletal dynamics and promote EMT-like processes, enhancing tumor cell migration [24,76]. Although direct evidence in sellar tumors remains limited, similar mechanisms are well established in other solid tumors [72,77]. In addition, EVs contribute to shaping the TME through the transfer of cytokines, growth factors, and regulatory RNAs, influencing stromal, endothelial, and immune cell populations. In craniopharyngiomas, characterized by a prominent inflammatory milieu, EVs may sustain pro-inflammatory signaling, while stromal-derived EVs further support tumor progression through bidirectional communication networks [38,48,78,79].

Angiogenesis represents another key EV-mediated process. The transfer of pro-angiogenic factors such as VEGF, together with regulatory miRNAs, promotes endothelial activation and neovascularization, facilitating tumor expansion within the sellar region [35,80]. Although metastatic dissemination is rare, EV-mediated signaling pathways involved in pre-metastatic niche formation may also be relevant in aggressive tumors [76].

Finally, EVs may influence therapeutic response and resistance. Changes in EV release and cargo composition have been observed following pharmacological treatments, including dopamine agonists used in PitNETs [81]. EV-mediated transfer of resistance-associated molecules could reduce treatment efficacy, whereas modulation of EV pathways may represent a novel therapeutic strategy [82]. Overall, EV-mediated communication integrates multiple functional processes central to sellar tumor biology. Despite limited direct evidence, current data support a model in which EV cargo links molecular alterations to tumor behavior and microenvironmental interactions [83].

**Figure 2:**
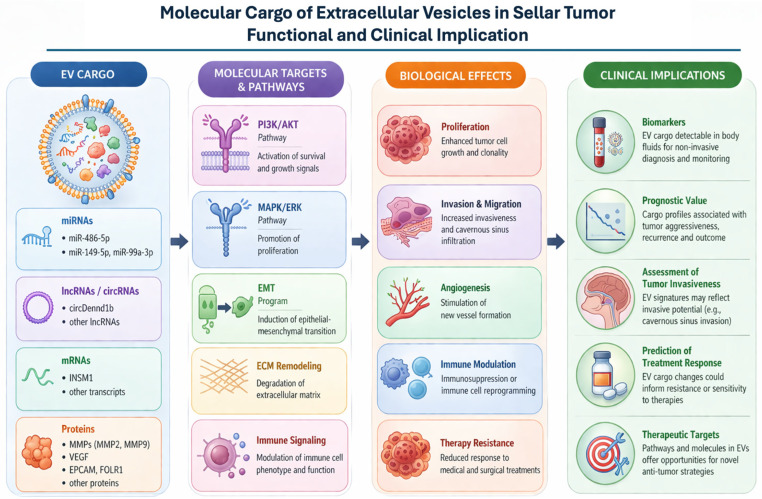
EVs derived from sellar tumors carry a diverse repertoire of biomolecules, including miRNAs, lncRNAs, circRNAs, mRNAs, and proteins. These cargo molecules modulate key signaling pathways, such as PI3K/AKT, MAPK, and EMT-related pathways. Through these mechanisms, EVs contribute to tumor proliferation, invasion, angiogenesis, immune modulation, and therapy resistance. These molecular features support the potential use of EVs not only as diagnostic and prognostic biomarkers in liquid biopsies, but also as therapeutic targets or innovative drug-delivery.

A schematic overview of the molecular cargo of EVs and their associated signaling pathways, biological effects, and clinical implications is provided in Figure 2.

## 5. EVs as Biomarkers in Sellar Tumors

The identification of reliable biomarkers for diagnosis, prognosis, and treatment monitoring remains a major unmet need in the management of sellar tumors [84]. Despite advances in imaging and hormonal assessment, predicting tumor behavior—particularly invasiveness, recurrence, and therapeutic response—continues to be challenging [4]. In this context, EVs have emerged as promising candidates for liquid biopsy, particularly in sellar tumors, where accessibility is limited and repeated sampling is rarely feasible [85].

One of the key advantages of EVs as biomarkers lies in their stability in biological fluids, including blood and CSF [9,10]. The lipid bilayer structure of EVs protects their molecular cargo from enzymatic degradation, allowing for the detection of nucleic acids and proteins that reflect the molecular characteristics of the originating tumor. In sellar tumors, circulating EVs may therefore provide insights into tumor subtype, biological activity, and progression. MiRNAs contained within EVs represent the most extensively investigated class of biomarkers in this setting [74,75]. Several studies have identified differential expression profiles of exosomal miRNAs in patients with PitNETs compared to healthy controls [47]. Specific miRNAs, such as miR-486-5p, have been proposed as potential diagnostic markers due to their consistent dysregulation in patient-derived samples [86]. However, the ability of EV-associated miRNAs to distinguish between invasive and non-invasive tumors remains less clear, with conflicting results reported across studies [32,43,58,87]. This variability likely reflects differences in cohort characteristics, analytical techniques, and EV isolation methods [88].

Beyond diagnosis, EV-associated cargo may also have prognostic value. The identification of molecular signatures associated with aggressive tumor behavior, including invasion of the cavernous sinus and resistance to therapy, could significantly improve patient stratification and management [4,35]. Preliminary data suggest that both RNA and protein components of EVs may correlate with tumor invasiveness, although these findings require validation in larger, well-characterized cohorts. Proteomic analysis of EVs offers an additional layer of biomarker potential. Differences in the expression of proteins such as EPCAM and FOLR1 have been reported in EVs derived from patients with invasive pituitary tumors, suggesting a possible role in identifying more aggressive disease phenotypes [15]. Similarly, EV-associated mRNAs reflecting neuroendocrine differentiation may serve as surrogate markers of tumor biology.

The use of CSF as a source of EVs represents an intriguing but underexplored avenue. Given the anatomical proximity of sellar tumors to the subarachnoid space, CSF-derived EVs may provide a more direct representation of tumor-related molecular signals compared to peripheral blood [89]. However, the invasiveness of CSF sampling and the limited availability of standardized protocols currently restrict its widespread application.

Despite their potential, several challenges must be addressed before EV-based biomarkers can be implemented in clinical practice. These include the lack of standardized methods for EV isolation and characterization, variability in analytical platforms, and the need for reproducibility across studies [10,20]. Furthermore, distinguishing TCdEVs from those released by normal tissues remains a significant challenge [20].

EV-based diagnostic and prognostic applications in sellar tumors should currently be regarded as exploratory. The available studies are generally limited by small cohorts, variable pre-analytical handling, heterogeneous isolation strategies, and inconsistent discrimination between invasive and non-invasive disease. Robust clinical implementation will require harmonized protocols, transparent reporting of EV characterization, external validation cohorts, and integration with established clinical, radiological, endocrine, and pathological parameters.

In summary, EVs represent a promising and rapidly evolving class of biomarkers in sellar tumors, with potential applications in diagnosis, prognosis, and treatment monitoring. While preliminary findings are encouraging, further validation and methodological standardization are essential to translate these insights into clinically useful tools.

## 6. Therapeutic Implications of Extracellular Vesicles in Sellar Tumors

The potential therapeutic applications of EVs in cancer have attracted significant interest in recent years; however, their translation into clinical practice remains at an early stage, particularly in the context of sellar tumors [26,79]. While most available evidence is derived from preclinical studies and from tumor types other than pituitary neoplasms, several conceptual strategies have been proposed that may be relevant to this field.

A possible approach involves targeting EV biogenesis, release, or uptake to disrupt tumor-promoting communication networks. Inhibition of secretion has been shown in experimental models to reduce tumor growth, angiogenesis, and metastatic potential [79,90]. Pharmacological agents interfering with EV production pathways, including inhibitors of neutral sphingomyelinase and vesicle trafficking components, have been explored in vitro and in vivo [91]. These strategies provide a rationale for targeting EV-mediated signaling in aggressive or treatment-resistant lesions.

Another widely explored strategy is the use of EVs as delivery vehicles for therapeutic agents [92]. Due to their biocompatibility and intrinsic ability to transfer molecular cargo, EVs have been proposed as natural nanocarriers for drugs and RNA-based therapies [93]. In central nervous system tumors, EVs have shown the ability to cross biological barriers and deliver functional cargo, suggesting potential applicability in anatomically complex regions such as the sellar area [94]. However, significant challenges remain, including loading efficiency, targeting specificity, and large-scale production.

From a translational perspective, potential sources of EVs could include mesenchymal stromal cell-derived EVs, immune cell-derived EVs, and engineered EVs generated from donor cells modified to express targeting ligands or an enriched therapeutic cargo. Candidate administration routes include systemic intravenous injection, intrathecal or intratumoral delivery during surgery. EV biodistribution and targeting may be assessed using fluorescence or bioluminescence imaging in preclinical models, magnetic resonance-compatible labeling strategies, or radionuclide-based approaches, although each modality has limitations in spatial resolution, sensitivity, and potential alteration of vesicle biology.

Modulation of EV cargo represents an additional potential strategy. Reintroduction of tumor-suppressive miRNAs through engineered EVs has shown anti-tumor effects in experimental models [95], and similar approaches have been associated with reduced proliferation and invasiveness in pituitary tumor cells [48].

Nevertheless, EV-based therapeutics face substantial translational barriers. These include scalable and reproducible manufacturing, batch-to-batch consistency, storage stability, cargo loading efficiency, targeting specificity, avoidance of unwanted biological effects, and compliance with regulatory requirements for complex biological products. These obstacles are particularly relevant in rare tumors such as sellar lesions, where preclinical models and disease-specific clinical trial infrastructures are limited.

EVs may also serve as tools to monitor treatment response and identify mechanisms of resistance [96]. Changes in EV cargo during therapy could provide insights into tumor adaptation and support treatment decision-making, particularly in PitNET where resistance to therapies such as dopamine agonists may occur. At present, no EV-based diagnostic or therapeutic approach has been clinically validated specifically for sellar tumors. Ongoing and early-phase EV-related clinical studies in oncology and neuro-oncology may nevertheless provide relevant information regarding feasibility, safety, biodistribution, and biomarker performance.

## 7. Technical Challenges and Limitations

Despite growing interest in EVs, several technical challenges limit their study and clinical translation, particularly in sellar tumors where available data are scarce [10,29,88]. A critical issue is that EV analysis is highly influenced by pre-analytical and analytical variables, including the biological fluid analyzed, sample collection procedures, processing time, centrifugation steps, and storage conditions. These factors may substantially affect EV yield, purity, molecular cargo preservation, and downstream reproducibility, thereby complicating comparisons across studies.

Several EV isolation and enrichment techniques are currently used in neuro-oncology and cancer-related studies, including differential ultracentrifugation, density-gradient centrifugation, size-exclusion chromatography, precipitation-based methods, ultrafiltration, immunoaffinity capture, and emerging microfluidic approaches. These methods differ substantially in yield, purity, scalability, reproducibility, and compatibility with downstream molecular analyses, making direct comparison across studies challenging [97]. A summary of commonly used EV isolation and characterization techniques, with their main advantages and limitations, is provided in Table 2.

Similarly, EV characterization requires a multiparametric approach, as no single method can fully define EV identity, purity, and functional relevance. Techniques such as nanoparticle tracking analysis, electron microscopy, Western blotting, flow cytometry, and omics-based analyses provide complementary information on EV size, concentration, morphology, surface markers, and molecular cargo. Therefore, orthogonal characterization strategies are essential, particularly in neuro-oncology studies where biological samples may contain mixed vesicle populations and non-vesicular contaminants [10]. In this context, standardization efforts such as the MISEV guidelines are particularly important for improving translational reproducibility. MISEV2023 emphasizes transparent reporting of EV source, sample handling, separation strategy, characterization methods, and analytical limitations, while discouraging overinterpretation of vesicle subtype identity when biogenesis-specific evidence is lacking. This is especially relevant for studies that use terms such as “exosomes” without direct demonstration of endosomal origin. Accordingly, throughout this review, the term EVs is preferentially used unless the cited studies specifically characterized exosomes or other EV subtypes according to established criteria.

These limitations are even more relevant in neuroendocrine and intracranial tumor contexts, where tumor-derived vesicles may be diluted within a large background of EVs released by blood cells, endothelial cells, inflammatory cells, and normal endocrine or neural tissues. In blood-based liquid biopsy studies, this biological background may reduce tumor specificity and complicate the interpretation of EV-associated molecular signatures. Although CSF-derived EVs could theoretically provide a more direct representation of tumor-related signaling in intracranial lesions, CSF sampling is invasive and current protocols remain limited by small sample volumes, variable recovery, and insufficient standardization [97,98]. Accordingly, future studies should integrate rigorous pre-analytical control, orthogonal EV characterization, and enrichment strategies for tumor-associated EV subpopulations, in line with MISEV2023 recommendations [10].

Because these approaches differ substantially in yield, purity, scalability, and compatibility with downstream molecular analyses, comparisons across studies remain difficult and reported findings may partly reflect methodological variability rather than true biological differences [97]. EV heterogeneity represents another critical issue. Current methodologies often fail to distinguish between vesicle subtypes with different biological functions, limiting the interpretation of experimental findings [17,20]. The identification of tumor-derived EVs within biological fluids remains challenging, as circulating EVs originate from multiple cell types, reducing specificity for liquid biopsy applications [99].

In addition, the relative paucity of data on EVs in sellar tumors limits the strength of current conclusions. Most available studies focus on pituitary neuroendocrine tumors, with minimal evidence in other lesions such as craniopharyngiomas. Furthermore, many findings are derived from in vitro models or extrapolated from other tumor types, which may not fully recapitulate the unique biological features of sellar tumors [100]. For this reason, EV-related mechanisms discussed in sellar tumors should be interpreted according to the level of available evidence, clearly distinguishing experimentally validated findings from hypothesis-generating extrapolations derived from other neuro-oncological or systemic tumor models.

## 8. Future Perspectives

The study of EVs in tumors of the sellar region is still in its early stages, yet it holds significant potential for advancing both biological understanding and clinical management [8,64,73]. Future research should move beyond descriptive profiling toward functional validation, with particular emphasis on establishing causal relationships between EV cargo and tumor behavior [10,88].

The development of advanced models, including patient-derived organoids, three-dimensional cultures, and in vivo systems, will be essential to investigate EV-mediated mechanisms in a physiologically relevant context. The adoption of consensus guidelines and the integration of multi-omics approaches, including transcriptomics, proteomics, lipidomics, and metabolomics, may facilitate a broader characterization of EV populations and their functional roles [101]. Emerging technologies such as single-EV analysis, microfluidic EV isolation, nanoscale flow cytometry, spatial transcriptomics, digital PCR, and AI-assisted biomarker discovery may further improve the sensitivity and biological interpretability of EV research in neuro-oncology and sellar tumors.

Another key direction involves the identification of tumor-specific EV signatures capable of improving patient stratification and supporting personalized therapeutic approaches [21]. In the context of pituitary neuroendocrine tumors, this could lead to improved patient stratification and more personalized treatment strategies. Integration of EV profiling with clinical, radiological, and computational data, including artificial intelligence-based models, may further enhance diagnostic and prognostic accuracy [102].

The role of the TME should also be further explored, particularly in relation to EV-mediated communication between tumor cells and stromal components [79]. Investigating the contribution of fibroblasts, immune cells, and endothelial cells to EV production and signaling may reveal novel mechanisms underlying tumor progression and resistance to therapy. This is especially relevant for tumors such as craniopharyngiomas, where inflammatory and cystic components play a central role [36].

Ultimately, large-scale prospective studies will be required to translate EV-based approaches into clinical practice and to validate their role in diagnosis, prognosis, and treatment monitoring [103]. By addressing these challenges, the field may progress from exploratory observations to clinically relevant applications, ultimately improving the management of patients with these complex tumors.

Future clinical translation will also require dedicated prospective trials or registry-based studies assessing EV-based biomarkers alongside conventional MRI, endocrine testing, ophthalmological outcomes, surgical findings, and histopathological classification. In the absence of sellar tumor-specific EV trials, lessons from ongoing EV studies in neuro-oncology and other solid tumors may help define appropriate endpoints, sampling schedules, analytical platforms, and safety requirements.

## 9. Conclusions

EVs have emerged as key mediators of intercellular communication in cancer, with increasing evidence suggesting their involvement in tumor behavior and microenvironmental interactions. In sellar tumors, including PitNETs and craniopharyngiomas, EV-mediated signaling represents a promising but still underexplored area of research. Current data suggest that EV cargo, including miRNAs, lncRNAs, mRNAs, and proteins, may contribute to fundamental processes such as tumor proliferation, invasiveness, angiogenesis, and immune modulation, although many mechanisms remain hypothesis-generating and require direct validation in sellar tumor models.

At the same time, EVs offer potential as minimally invasive biomarkers and, in the future, as therapeutic tools. A major challenge moving forward will be to translate these molecular insights into clinically meaningful applications. This will require standardized methodologies, robust validation in well-characterized patient cohorts, careful distinction between tumor-derived and background EV populations, and a deeper understanding of the functional impact of EV-mediated signaling in the unique niche of the sellar region.

In conclusion, EVs provide a compelling conceptual and translational framework for understanding tumor–microenvironment interactions in sellar tumors. While the field is still evolving, continued research integrating molecular, clinical, and imaging data has the potential to significantly advance both the biological understanding and clinical management of these complex lesions.

## Figures and Tables

**Table 1:** Summary of EV-associated cargo in sellar tumors, including functional roles, clinical implications, and supporting evidence.

Tumor Type	EV Source	Cargo	Biological Function	Implication	Level of Evidence	Reference
PitNET	Serum EVs	miRNA miR-486-5p	Differential exosomal expression in NFPA	Biomarker candidate	Clinical	Lyu et al., 2022 [47]
PitNET	Cell line EVs	miRNA miR-149-5p	Differential exosomal expression in NFPA	Functional/therapeutic candidate	Preclinical	Zhao et al., 2021 [48]
PitNET	Cell line EVs	miRNA miR-99a-3p	Inhibits progression (NOVA1/DTL/ RAB27B axis)	Functional/therapeutic candidate	Preclinical	Zhao et al., 2021 [48]
PitNET GH-secreting	Tumor- derived EVs	miRNA miR-21-5p	Promotes bone formation in acromegaly	Traslational proof-of-concept	Preclinical/translational	Xiong et al., 2020 [49]
Invasive non-functioning PitNET	Serum EVs	Protein FOLR1	Reduced in invasive NFPA	Marker of invasiveness	Clinical	Wang et al., 2019 [43]
Invasive non-functioning PitNET	Serum EVs	Protein EpCAM	Reduced in invasive NFPA	Marker of invasiveness	Clinical	Wang et al., 2019 [43]
PitNET	Serum EVs	mRNA-INSM1 mRNA	Increased serum mRNA-INSM1 in invasive PitNET	Marker of invasiveness	Preclinical/translational	Bao et a, 2020 [50]
Invasive non-functioning PitNET	Serum EVs	mRNA CDK6, RHOU	Identified in serum exosomes as biomarkers	Marker of invasiveness	Clinical/translational	Yu et al., 2019 [51]
PitNET	Serum EVs	circRNA circCCDC66	Proposed as diagnostic and prognostic serum exosomal circRNA	Biomarker candidate	Clinical/translational	Yue et al., 2023 [52]
PitNET microenvironment	CAF- derived EVs	circRNA circDennd1b	Promotes progression via miR-145-5p/ONECUT2 and MAPK	Stromal signaling and aggressiveness	Preclinical/mechanistic	Jiang et al., 2023 [53]
PitNET microenvironment	Serum EVs	Cargo protein MMP-related	EVs can traffic MMPs/MT1-MMP and facilitate matrix remodeling	Comparative evidence for invasiveness	Comparative/mechanistic	Thuault et al., 2022 [54]
PitNET microenvironment	Tumor- derived exosomes	VEGF	Promote angiogenesis	Comparative evidence for angiogenesis	Comparative/mechanistic	Ko et al., 2019 [55]
CFG (adamantinomatous)	Cyst fluid	IL-6, CXCL1, CXCL8, IL-10	Inflammatory microenvironment	Indirect evidence for EV-mediated signaling	Preclinical/translational	Donson et al., 2017 [56]
CFG (adamantinomatous)	Cyst fluid	POA1 and lipid-related proteins	Lipid metabolism pathways, inflammatory microenvironment	Biomarker candidate	Clinical/translational	Chen et al., 2024 [57]
Other Sellar tumors	Plasma/CSF EVs	Mixed cargo Multiple	Liquid biopsy potential	Future diagnostic potential	Conceptual/translational	Lisiewicz et al., 2024 [15]

**Table 2:** Overview of commonly used extracellular vesicle isolation and characterization techniques in neuro-oncology and cancer-related studies.

	Technique	Common Use	Advantages	Limitations
Isolation Techniques	Differential ultracentrifugation	EV enrichment from plasma, CSF, and other biological fluids	Widely used; suitable for large volumes; no chemical contamination	Time-consuming; operator-dependent; possible co-isolation of protein aggregates and lipoproteins
	Size-exclusion chromatography	Separation of EVs from soluble proteins	Improves purity and preserves vesicle integrity	Dilution of samples; limited ability to separate EV subtypes
	Polymer-based precipitation	High-yield EV enrichment from small volumes	Simple and rapid; requires limited equipment	Lower purity; frequent co-precipitation of contaminants
	Immunoaffinity capture	Enrichment of marker-defined EV subpopulations	Potentially improves tumor or cell-type specificity	Depends on marker selection; may miss heterogeneous EV populations
Characterization Techniques	Nanoparticle tracking analysis	Particle size and concentration assessment	Quantitative physical characterization	Limited phenotypic information; sensitive to contaminants
	Transmission electron microscopy	Morphological confirmation	Direct visualization of vesicle morphology	Low throughput; qualitative or semi-quantitative
	Western blot/ flow cytometry	Protein marker characterization	Supports assessment of EV-associated markers	Requires validated antibodies and careful controls
	RNA sequencing, proteomics	Cargo profiling	Enables biomarker discovery and multi-omics integration	Requires standardized normalization and validation cohorts

## Data Availability

No new data were created or analyzed in this study. Data sharing is not applicable to this article.

## Abbreviations

The following abbreviations are used in this manuscript:

BBB	blood–brain barrier
circRNAs	circular RNAs
CSF	cerebrospinal fluid
ECM	extracellular matrix
EMT	epithelial–mesenchymal transition
EPCAM	epithelial cell adhesion molecule
ESCRT	endosomal sorting complex required for transport
EVs	extracellular vesicles
FOLR1	folate receptor 1
INSM1	insulinoma-associated 1
lncRNAs	long non-coding RNAs
miRNAs	microRNAs
MMPs	matrix metalloproteinases
MRI	Magnetic Resonance Imaging
mRNAs	messenger RNAs
PitNETs	pituitary neuroendocrine tumors
TCdEVs	tumor cells-derived EVs
TME	Tumor microenvironment
VEGF	vascular endothelial growth factor
